# Heat shock protein DNAJA2 regulates transcription-coupled repair by triggering CSB degradation via chaperone-mediated autophagy

**DOI:** 10.1038/s41421-023-00601-8

**Published:** 2023-10-31

**Authors:** Yaping Huang, Liya Gu, Guo-Min Li

**Affiliations:** 1 Department of Radiation Oncology, University of Texas Southwestern Medical Center, Dallas, TX USA; 2 Chinese Institutes for Medical Research, Beijing, China

**Keywords:** Nucleotide excision repair, Lysosomes, Chaperone-mediated autophagy

## Abstract

Transcription-coupled nucleotide excision repair (TC-NER) is an important genome maintenance system that preferentially removes DNA lesions on the transcribed strand of actively transcribed genes, including non-coding genes. TC-NER involves lesion recognition by the initiation complex consisting of RNA polymerase II (Pol II) and Cockayne syndrome group B (CSB), followed by NER-catalyzed lesion removal. However, the efficient lesion removal requires the initiation complex to yield the right of way to the excision machinery, and how this occurs in a timely manner is unknown. Here we show that heat shock protein DNAJA2 facilitates the HSC70 chaperone-mediated autophagy (CMA) to degrade CSB during TC-NER. DNAJA2 interacts with and enables HSC70 to recognize sumoylated CSB. This triggers the removal of both CSB and Pol II from the lesion site in a manner dependent on lysosome receptor LAMP2A. Defects in DNAJA2, HSC70 or LAMP2A abolish CSB degradation and block TC-NER. Our findings discover DNAJA2-mediated CMA as a critical regulator of TC-NER, implicating the DNAJA2-HSC70-CMA axis factors in genome maintenance.

## Introduction

Nucleotide excision repair (NER) is a major DNA repair system that repairs bulky, helix-distorting lesions, including UV-induced cyclobutane pyrimidine dimers (CPDs) and 6-4 photoproducts, as well as environmental carcinogen-induced DNA lesions^1–4^. Defects in NER cause xeroderma pigmentosum (XP), an inherited skin cancer-prone disorder characterized by extreme sun sensitivity^5^. The NER reaction has been reconstituted in vitro using purified proteins, including seven factors known as XP complementation groups XPA to XPG^6,7^. There are two NER sub-pathways: global genome NER (GG-NER), which detects and repairs bulky lesions in the entire genome, and transcription-coupled NER (TC-NER), which specifically removes transcription-blocking lesions on the transcribed strand of transcriptionally active genes^1,8–11^, including non-coding genes^12,13^ that consist of 75%–80% of the human genome^14^. These two sub-pathways conduct lesion removal using the same core NER reaction, but differ in lesion recognition.

It is generally accepted that TC-NER is initiated with RNA polymerase II (Pol II) stalling by a transcription-blocking lesion on the transcribed strand of an actively transcribed gene. The stalled Pol II is recognized by the TC-NER-essential Cockayne Syndrome group B (CSB) protein, a central regulator of TC-NER^9–11,15,16^. The formation of this large DNA-protein complex facilitates the recruitment of downstream factors and assembly of the NER machinery to remove the DNA lesion^10,11,15^. However, for efficient lesion removal, it is critical to get rid of the lesion-stalled Pol II, because it shields the lesion^17,18^. Although direct dissociation of Pol II from the template strand^19^ and ubiquitination-dependent Pol II degradation^20,21^ have been observed during TC-NER, recent studies suggest that Pol II removal involves TFIIH-dependent displacement and Pol II backtracking^15,17,22–24^. However, CSB binds upstream of Pol II and pushes Pol II forward in a 3′-to-5′ direction over the DNA template strand^17,18^, which is the opposite direction of the Pol II 5′-to-3′ backtracking^10,17,18^. Thus, removing CSB from the recognition complex may allow Pol II to translocate in a backtracking manner, thereby promoting effective lesion removal. Previous studies have shown that CSB can be removed via proteasome-mediated degradation^25–29^ in response to UV treatment by Cockayne Syndrome group A (CSA)- or BRCA1-mediated polyubiquitination^25,26^, but a recent study demonstrated that CSA is required for removing sumoylated CSB in a manner independent of proteasome pathway upon UV irradiation^30^. Therefore, the mechanism that controls CSB’s timely removal and/or degradation during TC-NER is unclear.

Heat shock proteins (HSPs) play important roles in maintaining protein homeostasis in both normal and stressed conditions^31^, including UV-induced stress^32–34^. Interestingly, Ydj1, a member of the J-domain-containing HSP40 family in yeast, is required for cell survival, transcription recovery and NER efficacy in response to UV irradiation^35^. However, how Ydj1 is involved in transcription and NER is unknown. DNAJA2, the mammalian counterpart of Ydj1 that plays important roles in chromosomal stability^36^, likely plays a role in transcription and NER similar to Ydj1. Since HSP40 family proteins function as cochaperones of the 70 kDa HSP (HSP70) or the 71 kDa heat shock cognate (HSC70)^31–34^, we hypothesize that DNAJA2 is an important regulator for CSB in TC-NER.

Here, we show that DNAJA2 deficiency significantly reduces cell surviving ability and transcription recovery rate in response to UV irradiation. As a cochaperone of HSC70, DNAJA2 interacts with CSB and promotes CSB degradation through the HSC70 chaperone-mediated autophagy (CMA) pathway. Depleting DNAJA2 inhibits TC-NER activity, and is associated with delayed CSB degradation, persistent chromatin binding of CSB and Pol II. These observations suggest that DNAJA2 is involved in TC-NER by degrading CSB in a timely manner in response to UV-induced DNA damage on the transcribed strand of actively expressed genes. Therefore, this study has not only identified DNAJA2 as an essential factor for TC-NER, but also elucidated the molecular basis on which DNAJA2 regulates TC-NER by triggering CMA-catalyzed CSB degradation.

## Results

### DNAJA2-deficient cells are hypersensitive to UV and defective in TC-NER

**Fig. 1:**
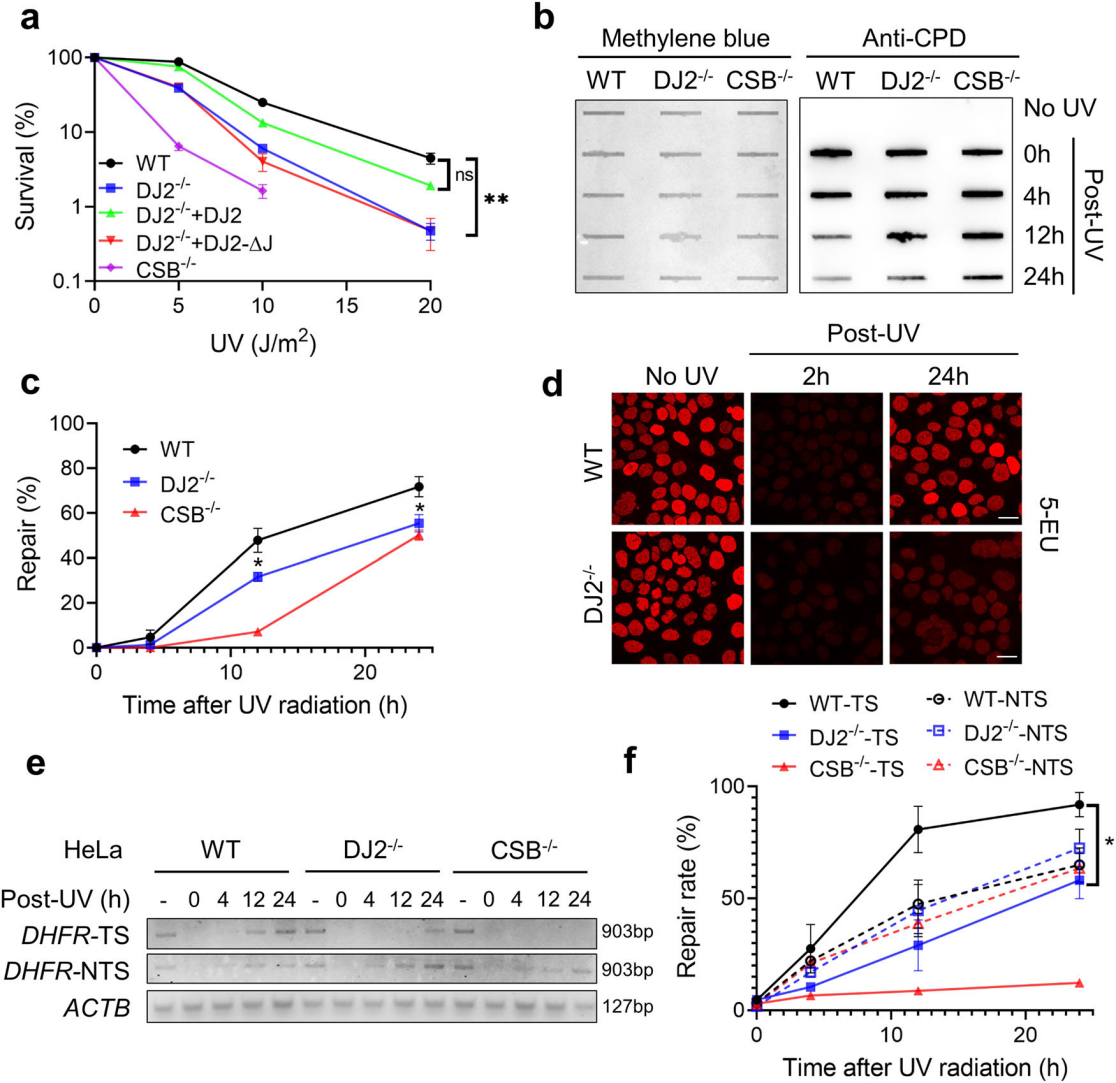
DNAJA2-deficient cells are hypersensitive to UV irradiation and defective in TC-NER. **a** Colony formation assay showing surviving fractions of WT HeLa, *DNAJA2* knockout (DJ2^−/−^) and knockouts rescued with WT DNAJA2 (DJ2^−/− ^+ DJ2) or J-domain truncated DNAJA2 (DJ2^−/− ^+ DJ2-ΔJ) in response to different doses of UV irradiation. *CSB* knockout (CSB^−/−^) cells were used as a control. Data from three independent experiments were used for the quantification and statistical test. **b** Slot blotting analysis detecting CPD levels in WT, DJ2^−/−^ and CSB^−/−^ HeLa cells at various time points post-UV irradiation (5 J/m^2^), as indicated, using an anti-CPD antibody. Methylene blue staining shows the total amount of DNA loaded in the assay. **c** Quantification of CPD removal efficiency (%) in WT, DJ2^−/−^ and CSB^−/−^ HeLa cells, as shown in **b**. CPD repair was calculated as: Repair (%) = (1 – CPD level of a given timepoint post UV/CPD level of 0 h post UV) 100%. Data from four independent experiments were used for the quantification and statistical test. **d** Immunofluorescence analysis showing transcriptional activity of WT and DJ2^−/−^ HeLa cells before and after UV irradiation (10 J/m^2^). Cells labeled with 5-EU were subjected to click reaction to conjugate a fluor probe. **e** Strand-specific PCR analysis to determine CPD removal in the transcribed (*DHFR-*TS) and non-transcribed (*DHFR-*NTS) strands of the *DHFR* gene at different time points after UV irradiation and digestion with T4 endonuclease V, which specifically nicks DNA at CPD sites. A fragment of *ACTB* gene containing no pyrimidine dimers was used as an internal control. **f** Quantifications of relative repair activities on TS and NTS in WT, DJ2^−/−^ and CSB^−/−^ HeLa cells. The relative repair efficiency was calculated by dividing the intensity of the PCR band of a given time point (0 h, 4 h, 12 h or 24 h) to that of without UV treatment (-). Data from three independent experiments were used for the quantification and statistical analysis. Scale bar, 20 μm. *P* values were determined by two-tailed unpaired *t*-test. ns, *P* > 0.05; *P < 0.05; **P < 0.01.

To explore the role of DNAJA2 in cellular response to DNA damage, we generated several *DNAJA2*-proficient and deficient HeLa cell lines, including *DNAJA2* knockout (KO) line (DJ2^−/−^), *DNAJA2*-rescued KO cell line (DJ2^−/−^+DJ2), and *DNAJA2-*KO line expressing the J domain-deleted DNAJA2 (DJ2^−/−^+DJ2-ΔJ) (Supplementary Fig. S1a). We determined the cell viability after treating cells with various doses of UV, which mainly induces cyclobutene pyrimidine dimers (CPDs) and 6-4 photoproducts. As shown in Fig. 1a and Supplementary Fig. S1b, DJ2^−/−^ cells were much more sensitive to UV irradiation than wild-type (WT) cells, which was similarly observed in CSB-KO (CSB^−/−^) cells. The sensitivity to UV in DJ2^−/−^ cells was reversed when the knockout cells expressed WT *DNAJA2* (DJ2^−/− ^+ DJ2), but not the J domain-deleted DNAJA2 (DJ2^−/− ^+ DJ2-ΔJ) (Fig. 1a). These observations indicate that the J domain of DNAJA2, which mediates its cochaperone activity of HSP40 proteins^31^, is essential for cellular response to UV-induced DNA damage.

To determine if DNAJA2 is involved in repair of UV-induced DNA lesions, we measured CPD levels using a CPD-specific antibody in DJ2^−/−^ and control HeLa cells after irradiating them with UV. The results showed that DJ2^−/−^ cells contained a CPD level, particularly at 12-h and 24-h time points, that is significantly higher than that in control cells (Fig. 1b, c). Immunofluorescence analysis also showed that the CPD removal rate was attenuated in *DNAJA2*-deficient (DJ2^−/−^ and DJ2^−/−^+DJ2-ΔJ) cells, as compared with *DNAJA2-*proficient (WT and DJ2^−/−^+DJ2) cells (Supplementary Fig. S1c, d). These phenomena are similar to those observed in CSB^−/−^ cells (Fig. 1b, c and Supplementary Fig. S1c, d). Collectively, these results suggest that DNAJA2 is essential for the removal of UV-induced DNA lesions.

To define the involvement of DNAJA2 in TC-NER or GG-NER, we analyzed the recovery of RNA synthesis in DJ2^−/−^ and control HeLa cells by directly visualizing the newly transcribed RNA after labeling with 5-ethynyl uridine (5-EU). As shown in Fig. 1d, nascent RNA synthesis was shut down at 2 h after UV irradiation in both WT and DJ2^−/−^ cells. However, RNA synthesis was completely recovered 24 h post-UV irradiation in WT cells, but the recovery was dramatically delayed in DJ2^−/−^ cells. These results indicate that the transcription recovery, which follows lesion removal by TC-NER^10,15,37^, is inhibited in the absence of DNAJA2. We therefore postulated that DNAJA2 is essential for TC-NER, whose timely removal of UV-induced lesions is critical for transcription recovery. To test this hypothesis, we measured the removal of CPDs in the dihydrofolate reductase (*DHFR*) gene, a well-studied locus for TC-NER^38–40^. Strand-specific PCR was performed to amplify the transcribed strand (TS) or non-transcribed strand (NTS) of a *DHFR* fragment^38^ after digestion with T4 endonuclease V, which specifically nicks DNA at CPD sites^41^. Thus, the level of the PCR products directly reports the repair activity on TS (TC-NER activity) and NTS (GG-NER activity). The results showed preferential repair of CPDs in TS over NTS in WT cells, as more repair products were observed in *DHFR-*TS than in *DHFR-*NTS 12 h and 24 h after UV irradiation (Fig. 1e, f). This preferential TC-NER repair is greatly reduced or disappeared in DJ2^−/−^ and CSB^−/−^ cells, respectively (Fig. 1e, f), suggesting that both DNAJA2 and CSB are involved in TC-NER. Although there was essentially no difference in PCR products from the NTS between WT and DJ2^−/−^ cells, significantly higher levels of the PCR products from the TS were detected in WT cells than in DJ2^−/−^ cells at each time point (Fig. 1e, f), indicating that DNAJA2 is required for TC-NER.

### DNAJA2-deficiency delays lysosome-mediated CSB degradation

**Fig. 2:**
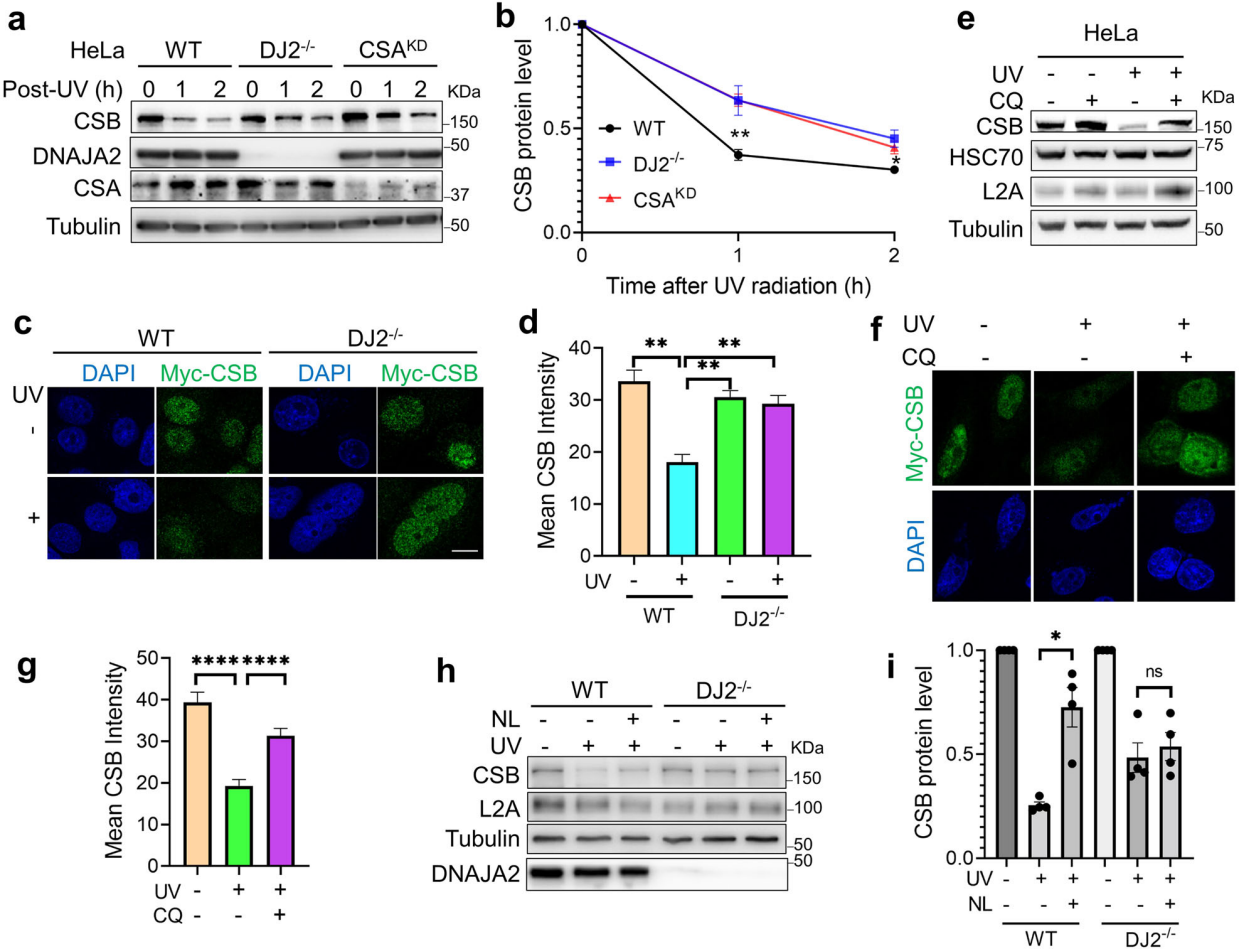
DNAJA2 deficiency delays UV-induced CSB degradation. **a** Western blotting analysis showing the CSB protein stability after UV irradiation (10 J/m^2^) in WT, DJ2^−/−^ and CSA knockdown (CSA^KD^) HeLa cells. **b** Quantification of the CSB protein levels in WT, DJ2^−/−^ and CSA^KD^ HeLa cells, as shown in **a**. Data from five independent experiments were used for the quantification and statistical analysis. **c** Representative images showing the protein levels of ectopically expressed myc-tagged CSB (myc-CSB) in WT and DJ2^−/−^ HeLa cells pre- or 1 h post-UV treatment (10 J/m^2^). **d** Quantifications of CSB intensities from 30–40 cells as shown in **c** by ImageJ. **e** Western blotting analysis showing the CSB protein stability in HeLa cells treated with or without UV in the presence or absence of a lysosome inhibitor CQ. Cells were pre-treated with 50 μM CQ for 4 h before UV irradiation (20 J/m^2^), and cultured for another 4 h before harvest in the presence of 50 μM CQ. **f** Representative images showing the myc-CSB intensity in HeLa cells treated with UV in the presence or absence of CQ. Cells were pre-treated with 50 μM CQ for 4 h before UV irradiation (10 J/m^2^), and cultured for another 2 h in the presence of CQ. **g** Quantifications of the CSB intensity, as shown in **f** by ImageJ. **h** Western blotting analysis showing the CSB protein stability after UV irradiation (10 J/m^2^) in the presence or absence of lysosomal protease inhibitors ammonium chloride and leupeptin (combined as NL), in WT and DJ2^−/−^ HeLa cells. Cells were pre-treated with 20 mM ammonium chloride and 100 μM leupeptin for 4 h before UV irradiation (10 J/m^2^), and cultured for another 2 h in the presence of NL. **i** Quantification of the relative CSB protein levels as shown in (**h**). Data from four independent experiments were used for the quantification and statistical analysis. Scale bar, 10 μm. *P* values were determined by two-tailed unpaired *t-*test. ns, *P* > 0.05; *P < 0.05; **P < 0.01; ****P < 0.0001.

Since HSP40 proteins function as cochaperones of HSP70/HSC70 in protein homeostasis^31,42–44^, we hypothesized that DNAJA2 participates in TC-NER by maintaining proteostasis of key TC-NER factor(s). To identify the factor(s), we directly compared the expression levels and turnover rates of key TC-NER components between WT and DJ2^−/−^ cells with or without UV irradiation. Among key TC-NER factors examined, which include CSA, CSB, Cul4, DDB1 and XPB, only CSB exhibited obviously UV-induced degradation in a DNAJA2-dependent manner (Fig. 2a, b and Supplementary Fig. S2a). Interestingly, CSB was degraded rapidly during the first 2 h post-UV treatment in WT cells (Fig. 2a, b), but this degradation was dramatically delayed in DJ2^−/−^ and CSA knockdown (CSA^KD^) cells (Fig. 2a, b and Supplementary Fig. S2b). While CSA is known to participate in the CSB degradation via the proteosome pathway^25^, our results here clearly suggest that DNAJA2 is also responsible for the CSB degradation in response to UV-induced DNA damage. Consistently, immunofluorescence analysis showed that the ectopically expressed myc-tagged CSB (myc-CSB) was also degraded slower in DJ2^−/−^ cells than in WT cells after UV irradiation (Fig. 2c, d).

HSP40 proteins are known to promote protein degradation through the lysosome-mediated autophagy pathway^31,42^. To test if the DNAJA2-regulated CSB degradation upon UV irradiation is dependent on the lysosomal pathway, we treated cells with chloroquine (CQ), an inhibitor of the lysosome degradation pathway. The results showed that CQ efficiently blocked the CSB degradation in all cell lines examined (Fig. 2e–g and Supplementary Fig. S2c), suggesting that the lysosomal pathway conducts the CSB degradation. To confirm that the lysosomal degradation of CSB is dependent on DNAJA2, we measured the CSB level in WT and DJ2^−/−^ cells treated with lysosomal protease inhibitors ammonium chloride and leupeptin (combined as NL) upon UV treatment. As shown in Fig. 2h, i, the NL treatment significantly stabilized CSB in WT but not DJ2^−/−^ cells. Taken together, these observations support the idea that DNAJA2 is responsible for the UV-induced CSB degradation via the lysosomal pathway.

### DNAJA2 promotes UV-induced CSB degradation via chaperone-mediated autophagy

**Fig. 3:**
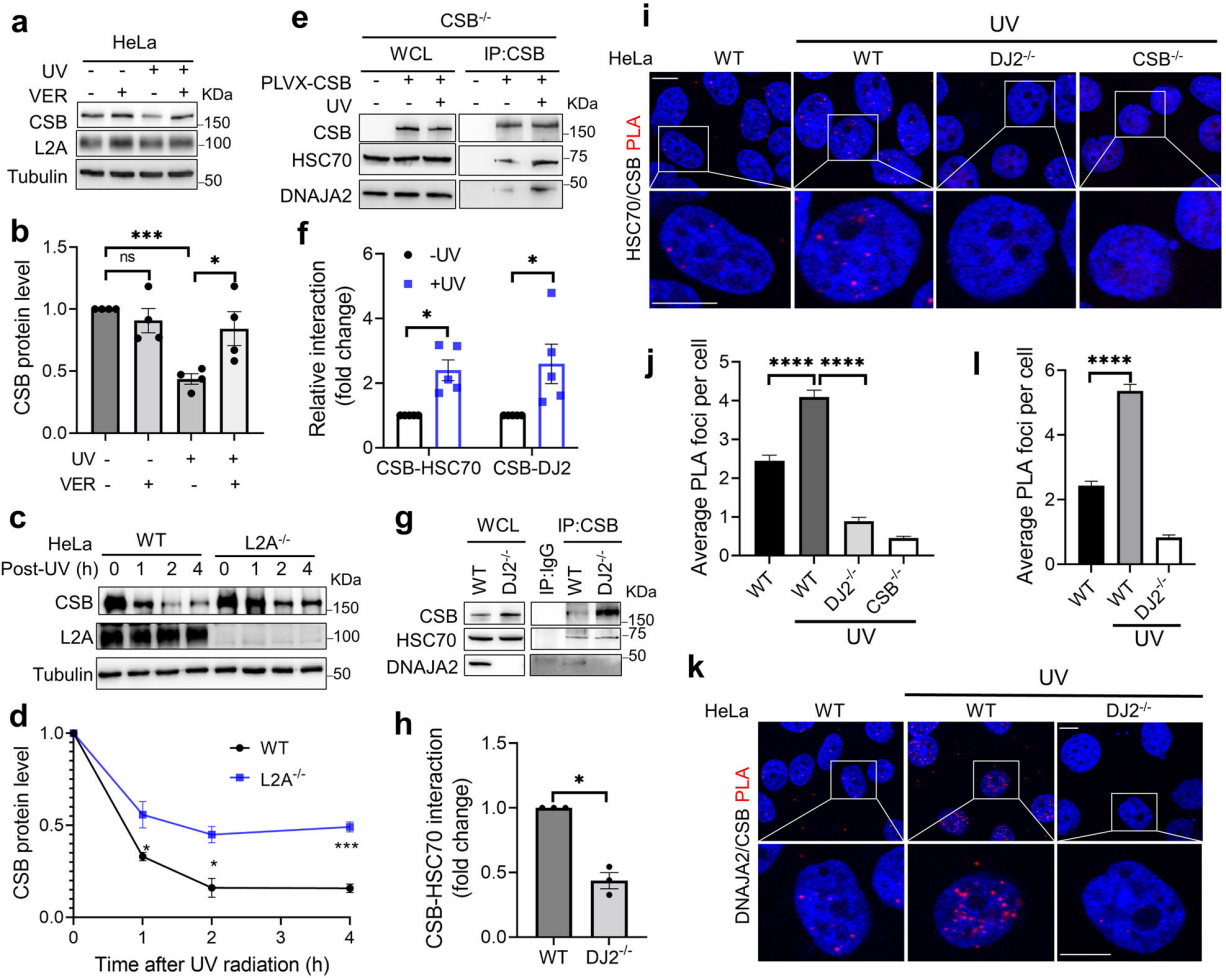
DNAJA2 promotes UV-induced CSB degradation via CMA pathway. **a** Western blotting analysis showing the CSB protein stability in HeLa cells treated with or without UV in the presence or absence of the HSC70 inhibitor VER-155008 (VER). Cells were pre-treated with 20 μM VER for 4 h before UV irradiation (10 J/m^2^), and cultured for another 1 h before harvest in the presence of 20 μM VER. **b** Quantification of the relative CSB protein levels as shown in **a**. Data from four independent experiments were used for the quantification and statistical analysis. **c** Western blotting analysis showing the CSB protein stability after UV treatment (10 J/m^2^) in WT and L2A^−/−^ HeLa cells. **d** Quantification of the CSB protein stability in WT and L2A^−/−^ HeLa cells, as shown in **c**. Data from three independent experiments were used for the quantification and statistical analysis. **e** Co-IP analysis showing UV-induced interaction between CSB and the HSC70/DNAJA2 chaperone complex. *CSB* knockout HeLa (CSB^−/−^) cells ectopically expressing CSB were treated with or without UV (20 J/m^2^) and harvested for IP assay 1.5 h after the treatment. **f** Quantification of the relative interaction between CSB and HSC70 (CSB-HSC70) or DNAJA2 (CSB-DJ2) in the presence (+UV) or absence (−UV) of UV irradiation, as shown in **e**. The fold change of relative interaction was calculated as: fold change = (IPed HSC70 or DNAJA2 intensity/IPed CSB intensity in +UV group) / (IPed HSC70 or DNAJA2 intensity/IPed CSB intensity in −UV group). Data from five independent experiments were used for the quantification and statistical analysis. **g** Co-IP analysis showing DNAJA2’s role in facilitating the HSC70-CSB interaction in response to UV treatment. WT and DJ2^−/−^ HeLa cells were harvested for IP assays 1.5 h after UV irradiation (20 J/m^2^). **h** Quantification of the relative interaction between CSB and HSC70 after UV treatment in WT and DJ2^−/−^ HeLa cells, as shown in **g**. Data from three independent experiments were used for the quantification and statistical analysis. **i** Representative PLA images showing UV-enhanced DNAJA2-facilitated CSB-HSC70 interaction. Cells were fixed and subjected to PLA analysis 1.5 h after UV irradiation (20 J/m^2^). CSB^−/−^ cells were used as a negative control. **j** Average PLA foci number in individual treatments from 180–260 cells, as shown in **i**. **k** Representative PLA images showing UV-enhanced DNAJA2-CSB interaction. Cells were fixed and subjected to PLA analysis 1.5 h after UV irradiation (20 J/m^2^). **l** Average PLA foci number in individual treatments from 220–310 cells, as shown in **k**. Scale bars, 10 μm. *P* values were determined by two-tailed unpaired *t*-test. ns, *P* > 0.05; *P < 0.05; ***P < 0.001; ****P < 0.0001.

Since DNAJA2 functions as a cochaperone of HSC70^43–45^, we postulated that the DNAJA2-facilitated CSB degradation is through the HSC70-mediated CMA pathway. To test this possibility, we first determined if HSC70 is essential for the UV-induced CSB degradation. We measured CSB stability in cells treated with UV in the presence of the HSC70 inhibitor VER-155008. As shown in Fig. 3a, b, inhibiting HSC70 indeed attenuated the UV-induced CSB proteolysis, suggesting that HSC70 is required for the DNAJA2-mediated CSB degradation after UV irradiation. We then knocked out *LAMP2A*, which encodes a lysosomal receptor essential for CMA, and analyzed CSB turnover. As observed in HSC70-inhibited cells, the UV-induced CSB degradation in *LAMP2A*-knockout (L2A^−/−^) cells was similarly delayed in comparison with control cells (Fig. 3c, d). These results suggest that CSB is a substrate of the DNAJA2-dependent HSC70-mediated CMA pathway. We also found that L2A^−/−^ cells were much more sensitive to UV treatment than WT cells (Supplementary Fig. S3a), and the CPD removal activity was dramatically inhibited in L2A^−/−^ cells (Supplementary Fig. S3b–d). These results suggest that DNAJA2-dependent HSC70-mediated CMA is essential for TC-NER.

We then performed co-immunoprecipitation (co-IP) analysis to determine direct interactions between CSB and the HSC70/DNAJA2 chaperone complex in the presence or absence of UV irradiation. The results showed that UV irradiation remarkably stimulated the interactions between CSB and the HSC70/DNAJA2 complex in both whole-cell lysates and chromatin fractions (Fig. 3e, f and Supplementary Fig. S4a, b), suggesting that UV irradiation enhances HSC70/DNAJA2-CSB interactions. Interestingly, despite abundant amount of CSB, significantly reduced CSB-HSC70 interaction was observed in DJ2^−/−^ cells (Fig. 3g, h), indicating that DNAJA2 facilitates the CSB-HSC70 interaction. The DNAJA2-dependent CSB-HSC70 interaction was further confirmed by the proximity ligation assay (PLA) (Fig. 3i–l). Taken together, the data shown here suggest that DNAJA2 is required for UV-induced CSB degradation by facilitating the CSB-HSC70 interaction.

**Fig. 4:**
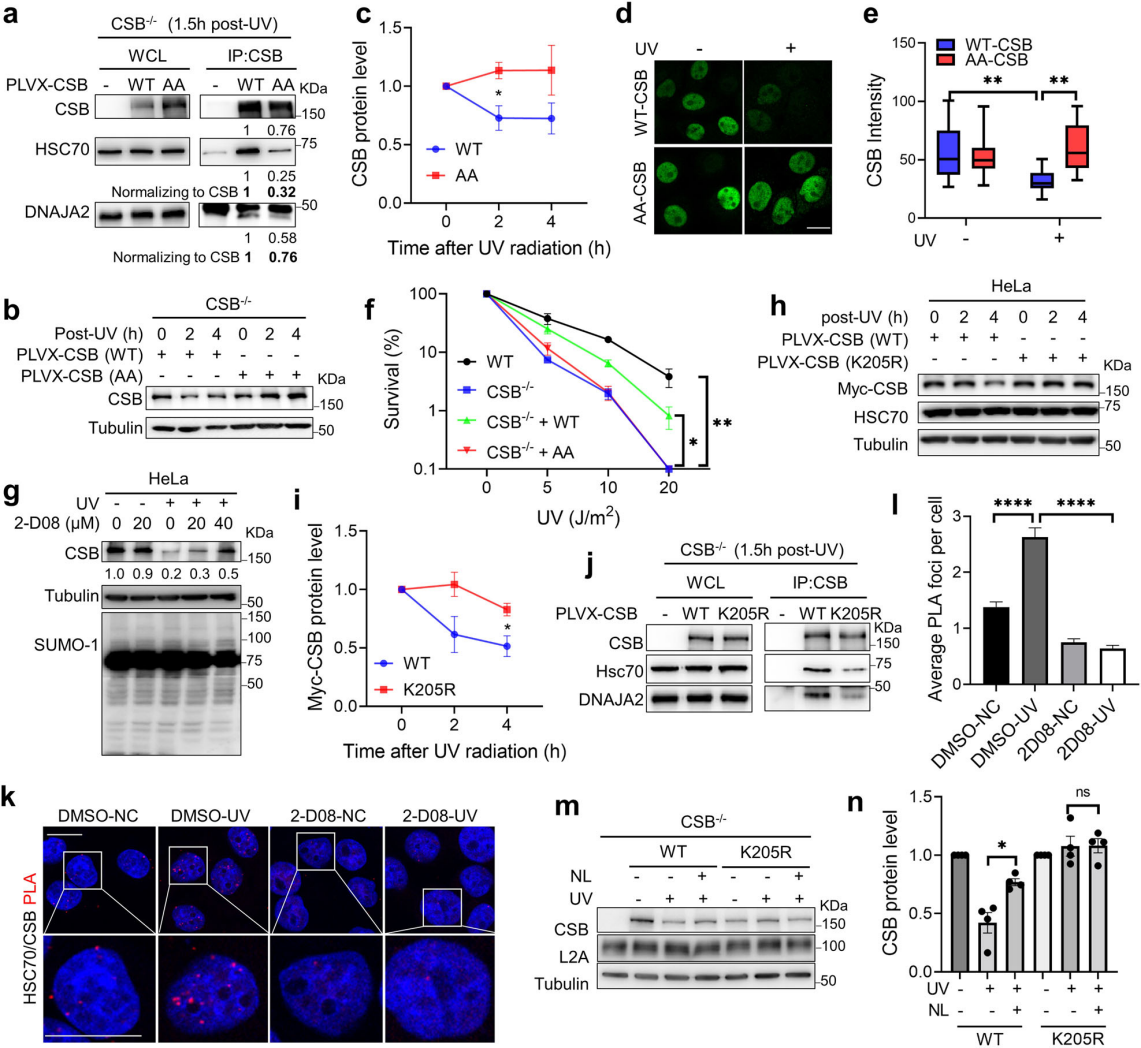
Sumoylated CSB is a bona-fide substrate of CMA. **a** Co-IP analysis showing involvement of the CSB KFERQ-like motif in CSB’s interactions with HSC70 and DNAJA2. Co-IP assays were performed in CSB^−/−^ HeLa cells expressing myc-tagged WT CSB or AA-CSB using an anti-CSB antibody after UV treatment (20 J/m^2^). **b** Western blotting analysis showing CSB stability in CSB^−/−^ cells expressing myc-tagged CSB or AA-CSB after UV irradiation (10 J/m^2^). **c** Quantification of the protein stability of CSB and AA-CSB after UV treatment, as shown in **b**. Data from three independent experiments were used for the quantification and statistical analysis. **d** Immunofluorescence analysis showing UV-induced degradation of CSB but not AA-CSB. myc-tagged CSB or AA-CSB was expressed in CSB^−/−^ HeLa cells and analyzed by immunofluorescence using an anti-myc tag antibody 1 h post UV treatment (10 J/m^2^). **e** Quantification of the CSB intensity by ImageJ (*n* = 31), as shown in **d**. **f** Colony formation assay showing surviving fractions of WT HeLa, CSB^−/−^ and knockouts rescued with WT CSB (CSB^−/− ^+ WT) or AA-CSB (CSB^−/− ^+ AA) in response to different doses of UV irradiation. **g** Western blotting analysis showing requirement of sumoylation in UV-induced CSB degradation in HeLa cells pre-treated with DMSO or a pan-sumoylation inhibitor 2-D08 for 6 h before UV irradiation (10 J/m^2^), followed by continuous culture for 1 h in the presence or absence of the inhibitor. **h** Western blotting analysis showing the stability of ectopically expressed CSB in HeLa cells expressing Myc-tagged WT CSB or CSB (K205R). **i** Quantification of the protein stability of WT CSB and CSB (K205R) after UV treatment, as shown in **h**. Data from three independent experiments were used for the quantification and statistical analysis. **j** Co-IP analysis showing involvement of the CSB K205 sumoylation in CSB’s interactions with HSC70 and DNAJA2. Co-IP assays were performed in CSB^−/−^ HeLa cells expressing Myc-tagged WT CSB or CSB (K205R) using an anti-CSB antibody after UV treatment (20 J/m^2^). **k** Representative PLA images showing the dependency of UV-induced HSC70-CSB interaction on CSB sumoylation. HeLa cells were pre-treated with DMSO or 40 μM 2-D08 for 6 h before UV irradiation (20 J/m^2^), followed by continuous culture for 1.5 h in the presence of DMSO or 2-D08. **l** Average number of PLA foci in individual treatments in 170–250 cells, as shown in **k**. **m** Western blotting analysis showing the protein stability of WT CSB and CSB (K205R) after UV irradiation (20 J/m^2^) in the presence or absence of lysosomal protease inhibitors NL. Cells were pre-treated with 20 mM ammonium chloride and 100 μM leupeptin for 4 h before UV irradiation (20 J/m^2^), and cultured for another 3 h in the presence of NL. **n** Quantification of the relative CSB protein levels as shown in **m**. Data from four independent experiments were used for the quantification and statistical analysis. Scale bars, 20 μm. *P* values were determined by two-tailed unpaired *t*-test. ns, *P* > 0.05; *P < 0.05; **P < 0.01; ****P < 0.0001.

CMA substrate usually contains a KFERQ-like motif to interact with the chaperone protein HSC70^42^. Indeed, we identified two potential KFERQ-like motifs in the human CSB sequence, i.e., the N-terminal _152_KIIEQ_156_ and the C-terminal _1482_REILQ_1486_ peptides (Supplementary Fig. S4c). Sequence analysis suggests that the N-terminal _152_KIIEQ_156_ motif is highly conserved across species (Supplementary Fig. S4d), implying its functional importance. We therefore substituted ‘EQ’ with ‘AA’ in the N-terminal motif (Supplementary Fig. S4c), and designated the mutant AA-CSB. The resulting mutant and WT CSBs were expressed in CSB^−/−^ cells and analyzed for their interactions with HSC70 by Co-IP after cells were treated with UV. As shown in Fig. 4a, WT CSB interacted with HSC70 much more efficiently than AA-CSB did, suggesting that the N-terminal KFERQ-like motif is involved in the interaction between CSB and HSC70.

To confirm the role of the CSB-HSC70 interaction in UV-induced CSB degradation during TC-NER, we analyzed the stability of WT CSB and AA-CSB. The results showed that WT CSB underwent degradation upon UV irradiation, but degradation of AA-CSB appeared to be undetectable (Fig. 4b, c). Immunofluorescence analysis also showed that AA-CSB was much more stable than WT CSB (Fig. 4d, e). Consistently, only WT CSB but not AA-CSB (Supplementary Fig. S4e) rescued the UV hypersensitivity of CSB^−/−^ cells (Fig. 4f). These results indicate that the KFERQ-like motif-mediated interaction between CSB and that HSC70 is required for UV-induced CSB degradation via the CMA pathway, which is essential for UV damage response and repair.

### Sumoylation of CSB triggers its UV-induced degradation by CMA

CSB has been reported to undergo UV-induced ubiquitination and subsequent degradation through the proteasome pathway^25–27^. However, a recent study showed that UV also induces CSB sumoylation, which is required for CSB removal^30^. Because UV treatment enhances the interaction between CSB and the HSC70/DNAJA2 chaperone complex (Fig. 3e, f and Supplementary Fig. S4a, b), we hypothesized that CSB sumoylation triggers its interaction with HSC70/DNAJA2, followed by degradation via the CMA pathway. To test this hypothesis, we first determined the UV-induced CSB degradation in cells treated with or without a sumoylation inhibitor 2-D08. As shown in Fig. 4g, the UV-induced CSB degradation was indeed inhibited by 2-D08 in a dose-dependent manner. We then created a mutated CSB by converting the key CSB sumoylation residue K205^30^ to R, which is near the CSB KFERQ-like motif (Supplementary Fig. S4c). We measured the UV-induced degradation of WT and K205R CSBs. The results showed that K205R-CSB was more stable than WT CSB protein in response to UV irradiation (Fig. 4h, i), suggesting that K205 sumoylation is required for UV-induced CSB degradation. To determine the impact of CSB sumoylation on its interaction with HSC70, we performed co-IP experiments in CSB^−/−^ cells expressing WT or K205R CSB after UV irradiation using an anti-CSB antibody. As shown in Fig. 4j, the K205R mutant displayed much weaker interaction with the DNAJA2/HSC70 chaperone complex. PLA analysis of HeLa cells treated with or without 2-D08 also showed that the UV-induced interaction between CSB and HSC70 was abolished when cells were treated with the sumoylation inhibitor (Fig. 4k, l), suggesting that CSB sumoylation promotes its interaction with HSC70, followed by its degradation.

To confirm that the sumoylated CSB undergoes lysosomal degradation via the CMA pathway, we analyzed UV-induced degradation of WT and sumoylation-deficient K205R CSB by treating CSB^−/−^ cells expressing WT or K205R CSB with lysosomal proteases inhibitors NL. As shown in Fig. 4m, n, the NL treatment significantly blocked UV-induced degradation of WT but not K205R CSB. Taken together, the DNAJA2/HSC70 chaperone complex targets sumoylated CSB for lysosomal degradation via the CMA pathway.

### Timely removal of CSB by DNAJA2-mediated CMA is essential for TC-NER

**Fig. 5:**
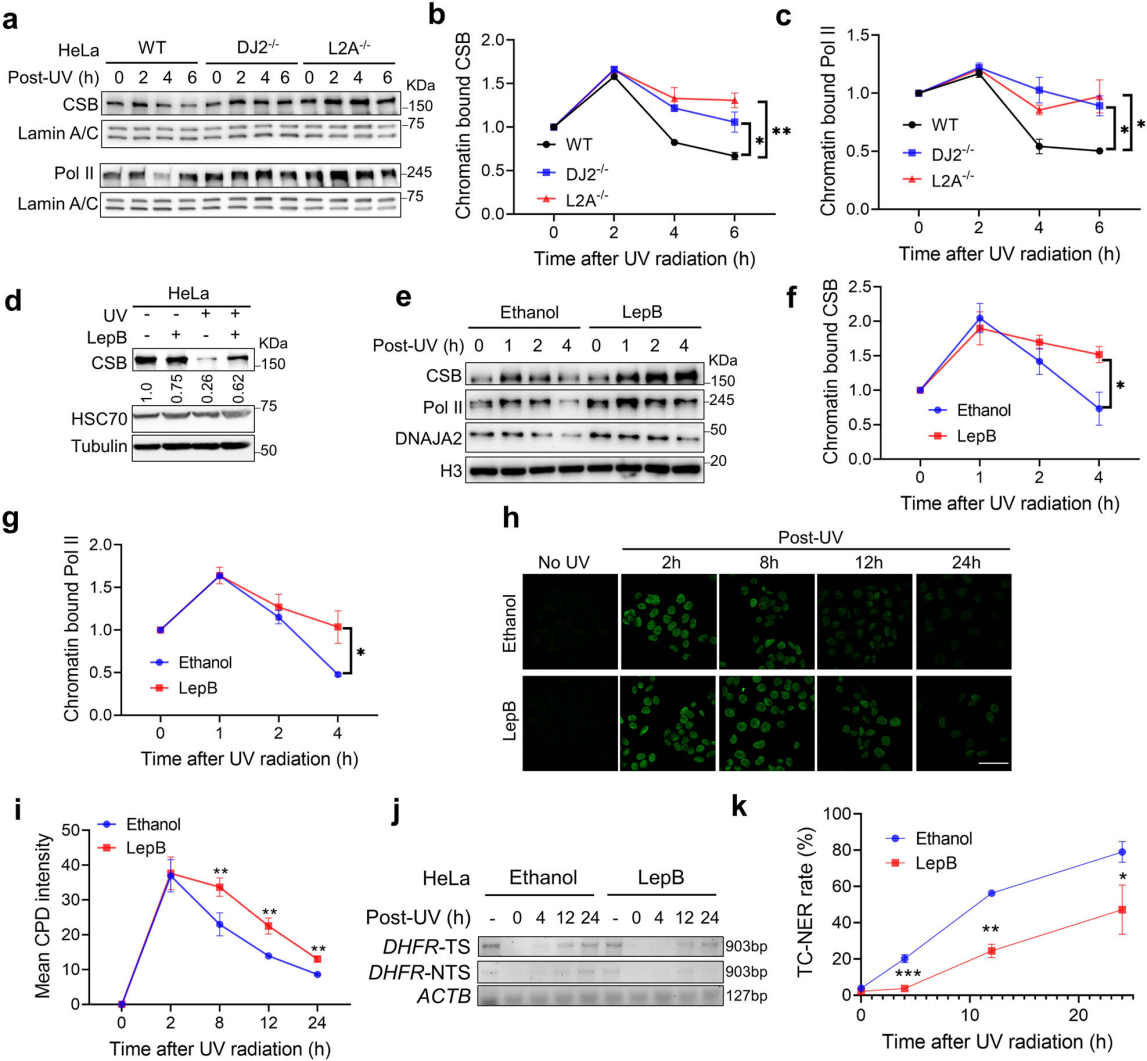
Timely removal of CSB by DNAJA2-facilitated CMA is essential for TC-NER. **a** Western blotting analysis showing the dynamic changes in the protein levels of CSB and Pol II in chromatin fractions after UV treatment (20 J/m^2^) in WT, DJ2^−/−^ and L2A^−/−^ HeLa cells. **b**, **c** Quantifications of the chromatin-bound CSB (**b**) and Pol II (**c**), as shown in **a**. Data from three independent experiments were used for quantification and statistical analysis. **d** Western blotting analysis showing the blockage of UV (20 J/m^2^)-induced CSB degradation by nuclear export inhibitor LepB. **e** Western blotting analysis showing UV-induced dynamic changes in protein levels of CSB and Pol II in chromatin fractions in LepB-treated or untreated HeLa cells. Cells were pre-treated with 20 nM LepB for 2 h before 20 J/m^2^ UV irradiation and continuously cultured for the indicated times in the presence or absence of LepB. **f**, **g** Quantifications of the dynamic changes of chromatin bound CSB (**f**) and Pol II (**g**), as shown in **e**. Data from three independent experiments were used for quantification and statistical analysis. **h** Immunofluorescence analysis of CPD removal after UV irradiation (5 J/m^2^) in HeLa cells treated with LepB or ethanol. **i** Quantification of the mean CPD intensity shown in **h**. Data from three independent experiments were used for quantification and statistical analysis. **j** Strand-specific PCR analysis to determine CPD removal after UV irradiation (5 J/m^2^) in the transcribed (*DHFR-TS*) and non-transcribed (*DHFR-NTS*) strands of the *DHFR* gene at different time points after UV irradiation in HeLa cells treated with LepB or ethanol. A fragment of *ACTB* gene without pyrimidine dimers was amplified as an internal control. **k** Quantification of relative TC-NER activity, as shown in **j**. For each group (Ethanol or LepB), the relative TC-NER efficiency was calculated by dividing the intensity of the PCR band of a given time point (0 h, 4 h, 12 h, 24 h) to that without UV treatment (-). Data of three independent experiments were used for the quantification and statistical analysis. For experiments in **h**–**k**, cells were pre-treated with LepB or ethanol for 4 h before UV irradiation, and further cultured for 1–4 h in the presence of the chemical. Cells were then maintained in fresh medium as needed. Scale bar, 50 μm. *P* values were determined by two-tailed unpaired *t*-test. *P < 0.05; **P < 0.01; ***P < 0.001.

Based on the current model, the efficient lesion removal by TC-NER requires backtracking of the lesion-bound Pol II, but CSB, which forms an initiation complex with Pol II, acts as a roadblock for Pol II’s backtracking^17,18^. Therefore, removal of CSB upon assembly of the TC-NER complex has been proposed to be essential for TC-NER. We proposed that the DNAJA2/HSC70-involved CMA pathway plays a role in the timely CSB removal from the initiation complex. If this were true, cells defective in the DNAJA2/HSC70-CMA axis would possess a stable chromatin-bound level of CSB, thereby a stable Pol II as well, after UV treatment. To test these possibilities, we firstly performed chromatin fractionation assay to determine the dynamics of chromatin-bound CSB in UV-treated WT, DJ2^−/−^ and L2A^−/−^ HeLa cells. The results showed that the level of chromatin-bound CSB gradually decreased in WT cells 2 h post-UV irradiation, but this decrease was significantly slower in DJ2^−/−^ and L2A^−/−^ cells (Fig. 5a, b). As expected, the level of Pol II trapped on chromatin was higher in DJ2^−/−^ and L2A^−/−^ cells than in control cells (Fig. 5a, c). These results support the idea that DNAJA2-mediated CMA is responsible for CSB degradation and subsequently Pol II release from the damage site to facilitate the assembly of the excision machinery.

Since the CMA-catalyzed degradation of CSB requires CSB to translocate from nucleus to cytosol, we postulated that blocking such translocation inhibits TC-NER. To test this hypothesis, we treated HeLa cells with the nuclear export inhibitor leptomycin B (LepB) and measured the CSB level after UV irradiation. The results showed that the nuclear CSB level was significantly higher in LepB-treated cells than in untreated control cells (Fig. 5d and Supplementary Fig. S5a–c), indicating that the UV-induced degradation of CSB is largely inhibited when protein nuclear export is blocked. As expected, the LepB treatment had less effect on the stability of CSB in DJ2^−/−^ cells (Supplementary Fig. S5a), further suggesting the involvement of DNAJA2 in CSB proteolysis. Taken together, these observations strongly indicate that CSB is translocated from nucleus to cytosol for degradation via DNAJA2-mediated CMA upon UV irradiation.

To determine if LepB treatment traps CSB on chromatin, which in turn blocks Pol II release from damage sites, thereby inhibiting TC-NER, we analyzed the chromatin-bound CSB and Pol II in LepB-treated and untreated cells in response to UV treatment. As shown in Fig. 5e–g, more CSB and Pol II were detected on chromatin in LepB-treated cells than in control cells 2 h after UV treatment. To test if the chromatin-trapped CSB and Pol II impair DNA damage response and TC-NER, we treated cells with LepB and measured overall CPD removal and TC-NER activity after UV irradiation. We found that in comparison to untreated cells, LepB-treated cells exhibited a significant delay in the CPD removal (Fig. 5h, i) and much lower TC-NER activity (Fig. 5j, k). Taken together, these results demonstrate that timely translocation of CSB from nucleus to cytosol for lysosome-mediated degradation is essential for TC-NER.

## Discussion

TC-NER is an important genotoxicity avoidance system that repairs DNA helix-distorting lesions resulting from environment and cellular metabolites during transcription^1,9–11^. It is well accepted that the repair event starts with lesion recognition by RNA Pol II in coordination with CSB, but the initiation/recognition complex must be taken away from the damage site to yield the right of way to the subsequently assembled excision machinery that carries out the lesion removal. However, how the recognition complex is timely removed is unclear. In this study, we provide strong evidence showing that the heat shock protein DNAJA2 functions as a cochaperone of HSC70 to promote UV-induced CSB degradation through the CMA pathway, thus removing Pol II from the lesion site for lesion verification and removal by the downstream NER factors.

**Fig. 6:**
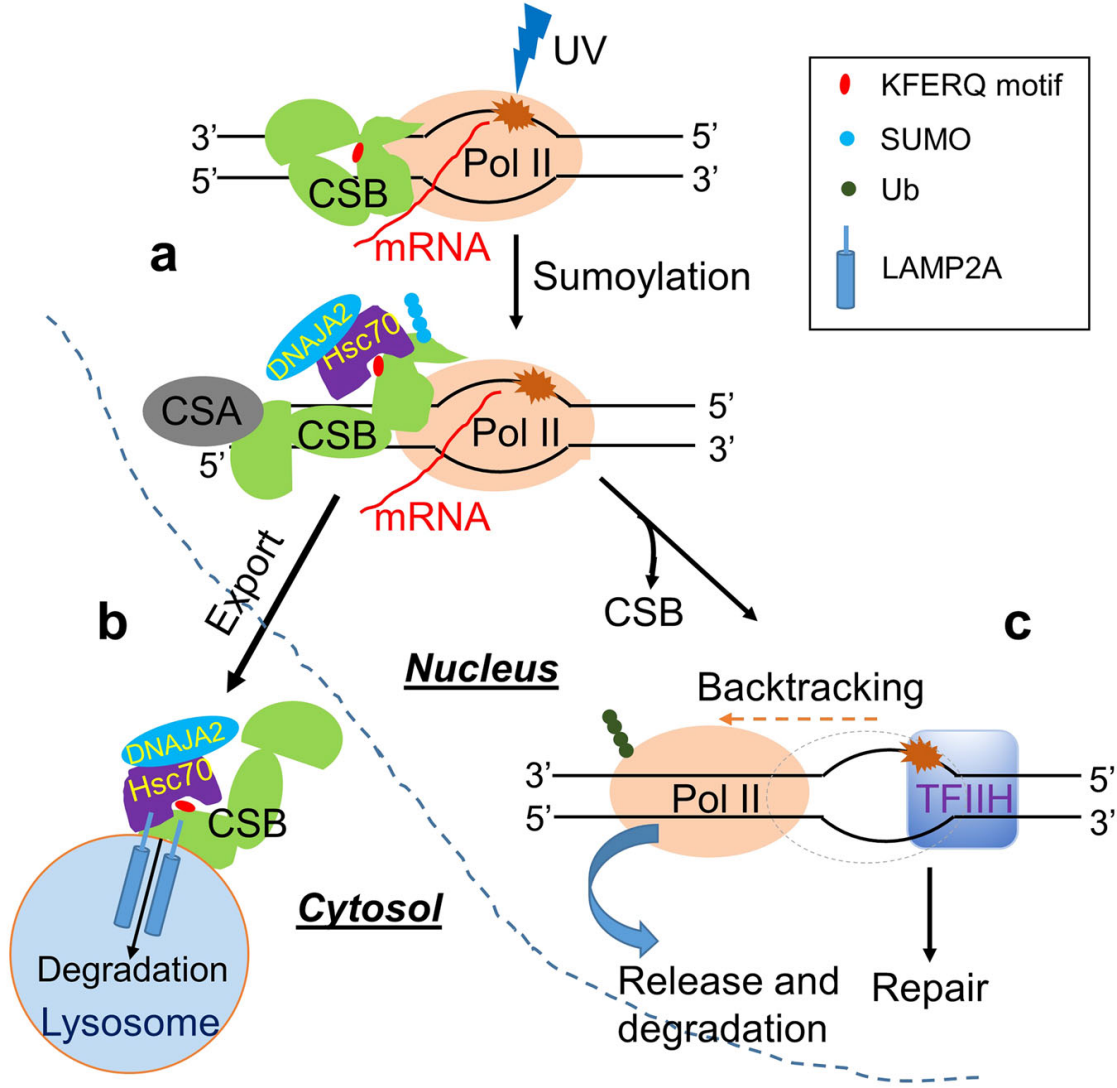
A working model proposing how the DNAJA2/HSC70 chaperone complex regulates CSB proteolysis and TC-NER. Upon UV irradiation, a transcription-blocking lesion is recognized by Pol II, which recruits CSB and CSA to the lesion to facilitate lesion recognition and stimulates sumoylation of CSB at its N-terminal domain. This sumoylation leads to a conformation change in CSB, making its KFERQ-like motif accessible to the DNAJA2/HSC70 chaperone complex, which binds to CSB (**a**). The DNAJA2/HSC70 complex exports CSB from nucleus to cytosol, where CSB undergoes lysosome degradation through LAMP2A-mediated CMA (**b**). The timely removal and degradation of CSB from the lesion permits Pol II to undertake a 5′-3′ translocation, also called backtracking, which makes the lesion available for verification by the downstream factor TFIIH and repair complex assembly to complete TC-NER (**c**).

Based on previously published data and the results presented here, we propose a working model for the critical role that DNAJA2 plays in TC-NER by triggering timely degradation of CSB via HSC70-mediated CMA (Fig. 6). During lesion recognition, CSB is located upstream of Pol II and pushes Pol II forward in a 3′-to-5′ direction over the DNA template strand^17,18^. To facilitate Pol II backtracking, a 5′-to-3′ movement along the DNA template strand, the backtracking blocker CSB is sumoylated at its N-terminal domain^30^, which triggers its conformation change to expose the KFERQ-like motif. This promotes physical interaction between CSB and the HSC70/DNAJA2 chaperone complex (Fig. 6a). This interaction is critical for directing CSB degradation through LAMP2A-mediated CMA (Fig. 6b), as disrupting the CSB’s interaction with the HSC70/DNAJA2 complex inhibits UV-induced CSB degradation and eventually TC-NER (Figs. 3 and 4). Upon the removal of the roadblock CSB from the recognition complex, Pol II undergoes backtracking, which releases the lesion for cleavage by NER factors including TFIIH (Fig. 6c). We believe that in this process DNAJA2 forms a chaperone complex with HSC70 to transport CSB from the nucleus to cytoplasm for CMA-conducted degradation in response to UV irradiation.

Both CRL^CSA^-mediated^25^ or BRCA1-dependent^26^ ubiquitination and USP7-mediated deubiquitination^28,29,46^ of CSB have been reported to coordinate CSB homeostasis and dynamics at the damage site after UV irradiation^10,15,16,23^. Here, we show that inhibition of the lysosome pathway or protein sumoylation prevents UV-induced CSB degradation. These observations are consistent with recent studies showing that CSB is sumoylated at its N-terminal domain in response to UV irradiation, and that this sumoylation is essential for coordinating the CSB homeostasis and TC-NER^30,47^. It is possible that different post-translational modifications (sumoylation and CSA-mediated ubiquitination) contribute to CSB proteostasis in different steps or subreactions of TC-NER. Although the mechanism by which sumoylation affects CSB stability in response to UV irradiation remains to be determined, CSA seems to play an important role in the clearance of sumoylated CSB in a manner independent of the proteasome pathway^30^. CSA directly interacts with chaperone complex TRiC, and regulates the assembly of CSA-mediated CRL^CSA^ E3 ligase complex and TC-NER activity^48^. CSA may function as a scaffold protein to interact with and recruit other chaperone proteins to the damage site, which facilitates the removal of CSB. Consistent with this possibility, we found that the HSC70/DNAJA2 chaperone complex strongly binds to CSB in response to UV irradiation and promotes CSB dissociation from the chromatin and subsequent degradation via the CMA pathway. Whether or not CSA facilitates HSC70/DNAJA2-mediated CSB degradation is worth investigating.

We show that the interaction between the HSC70/DNAJA2 complex and CSB is remarkably enhanced by UV irradiation (Fig. 3), and that the CSB protein level under normal condition is not affected by the chaperone complex (Figs. 2a and 3a), suggesting that CSB may change its protein conformation to expose the KFERQ-like motif in response to UV irradiation. It has been proposed that CSB undergoes a conformational change, which may open its N-terminal domain for specific interaction with the HSC70/DNAJA2 complex after lesion recognition^49,50^. Interestingly, the most C-terminal domain is essential for CSB’s sumoylation at its N-terminal domain and TC-NER activity^47^, suggesting that the proximity or interaction between the N-terminal domain and most C-terminal region is required for CSB sumoylation when CSB is in its closed conformation. However, it is the sumoylation that alters the structure of CSB, making it accessible to the DNAJA2/HSC70-mediated CMA. Therefore, this study has discovered a novel mechanism that regulates TC-NER, i.e., the heat-shock protein DNAJA2-mediated CMA. Given that defects in TC-NER cause cancer and premature aging, we predict that deficiency in DNAJA2 could have similar impact on human diseases. It has been documented that DNAJA2 plays an important role in cancer development and therapy. Whether or not this is related to its role in regulating TC-NER remains to be investigated.

In this study, we identified CMA pathway as a novel cytosolic pathway that regulates the nuclear TC-NER. Consistent with a previous study showing that the DNA damage response kinase Chk1 is degraded via the CMA pathway^51^, we identified CSB as another nuclear protein that undergoes CMA-mediated degradation, suggesting that nuclear proteins may be common clients for the CMA pathway. Defects in CMA or inhibition of the HSC70 activity retains CSB in the nucleus but not in the cytosol, implying that the client recognition and translocation step and the lysosomal degradation step are well-coordinated and regulated. It is possible that the chaperone complex senses intact CMA status and communicates between nuclear proteins and the CMA machinery. However, the detailed mechanism by which the CMA pathway targets nuclear proteins for degradation requires further investigation.

## Materials and methods

### Plasmids, reagents and antibodies

DNAJA2 cDNA (full-length or J-domain truncated) was cloned into the pLenti vector. The WT PLVX-myc-CSB plasmid was a gift of Dr. Li Lan (Harvard Medical School Massachusetts General Hospital). CSB mutant plasmids expressing CSB (E_155_Q_156_-AA) and CSB (K205R) were generated from the WT one by site-directed mutagenesis. Plasmid transfections were performed using jetPRIME® Transfection reagent (PolyPlus, #114-07).

Staining dyes used in this study include ProLong™ Diamond Antifade Mountant with DAPI (Molecular Probes™, P36962), methylene blue (Sigma, M9140) and crystal violet (Sigma, C0775), Click-iT™ RNA Alexa Fluor™ 594 Imaging Kit (Invitrogen C10330). Compounds including Chloroquine (HY-17589A), Leupeptin (HY-18234A), VER-155008 (HY-10941), Leptomycin B (HY-16909) and 2-D08 (HY-114166) were purchased from MedChemExpress.

Antibodies used were purchased from commercial resources: anti-DNAJA2 (Sigma, WH0010294M1); anti-CSA (GeneTex, GTX100122); anti-LAMP2A (ab18528), anti-DNAJA2 (ab157216) and anti-CSB (ab96089) from abcam; anti-DNAJA2 (12236-1-AP), anti-HSC70 (10654-1-AP) and anti-Lamin A/C (10298-1-AP) from proteintech; anti-α-tubulin (B-7) (sc-23948), anti-HSC70 (B-6) (sc-7298), anti-SUMO-1 (D-11) (sc-5308), anti-CSA (D-2) (sc-376981), anti-CUL4 (H-11) (sc-377188), anti-DDB1 (E-11) (sc-376860) and anti-β-Actin (C4) (sc-47778) from Santa Cruz; anti-Phospho-Rpb1 CTD (Ser2) (E1Z3G) (#13499), anti-Rpb1 CTD (4H8) (#2629) and anti-Histone H3 (D1H2) XP® (#4499) from Cell Signaling; anti-myc-tag (AE010) from Abclonal; anti-Cyclobutane Pyrimidine Dimers (CPDs) mAb (Clone TDM-2) (Cosmo Bio, CAC-NM-DND-001).

### Cells and cell culture

Unless specified, all cells were maintained at 37 °C and 5% CO_2_. All gene knockout cell lines were generated using the CRISPR-Cas9 system^52^. HeLa cells were cultured in RPMI 1640 media supplemented with 10% FBS; SV-589, B16-OVA and 4T1 cells were grown in Dulbecco’s Modified Eagle Medium (DMEM) supplemented with 10% FBS.

When treated with UV, cells were washed with PBS twice and subjected to global UVC irradiation at the indicated doses. When indicated, cells were treated with 50 μM Chloroquine (CQ), 20 mM ammonium chloride and 100 μM leupeptin (combined as NL), 20 μM VER-155008 or 20 nM Leptomycin B, respectively, for 2–6 h before UV irradiation, and continuously cultured in the presence of the specified chemical for the indicated time points before harvest.

### Colony formation assay

Two thousand cells were plated in each well of a 6-well plate for 24 h before treated with various doses of UV for 48 h. The treated cells were cultured in fresh media for another 10 days to allow colony formation. Colonies were stained with 0.25% crystal violet in 70% ethanol/PBS solution and counted. The surviving colonies were normalized to the colony formation efficiency, which was calculated by dividing the colony number in the un-irradiated group to the number of total cells plated in the well.

### CPD slot blotting analysis

The CPD measurement assay was performed as described^53^. Briefly, genomic DNA (gDNA) was isolated from cells subjected to 5 J/m^2^ UVC irradiation and denatured by heating. The gDNA (100 ng) was applied onto a Hybond nitrocel-lulose membrane assembled in a slot blot apparatus. Another replicate of all samples was loaded on the membrane side-by-side, which served as loading controls. The membrane was then baked in a vacuum oven at 80 °C for 1 h to crosslink the DNA onto the membrane, and subsequently cut into two pieces to separate the two replicates. One replicate was immediately stained with 0.3% methylene blue. The other replicate was blocked in 5% milk at room temperature for 1 h and incubated with anti-CPD antibody at 4 °C overnight, followed by secondary antibody incubation and imaging using ChemDoc.

### Nascent RNA labeling

WT and DJ2^−/−^ HeLa cells were incubated in medium containing 5-ethynyl uridine (5-EU) for 1 h and the EU-labelled nascent RNA was detected using the Click-iT RNA Imaging kits (Invitrogen) according to the manufacture instructions. Images were acquired using a Leica TCS SP8 confocal microscope.

### TC-NER activity assay

The TC-NER activity was determined using the strand-specific PCR assay as described^38^. Briefly, gDNA isolated from cells without UV treatment or at different time points (0, 4, 12 and 24 h) post UV irradiation was digested with T4 endonuclease V (Endo V), which specifically recognizes and cleaves DNA at CPD sites. Specific primers for transcribed and non-transcribed strands of *DHFR* gene were used for first-strand PCR and the subsequent strand-specific PCR. A fragment of the *ACTB* gene containing no pyrimidine dimers was used as an internal control.

### Indirect immunofluorescence

Cells grown on the cover slides were fixed with 4% paraformaldehyde for 10 min at room temperature and subsequently permeabilized with 0.25% Triton X-100 for 10 min. The slides were incubated with 5% BSA for 30 min at room temperature, followed by primary and secondary antibody incubation before mounting with DAPI medium. For CPD staining, DNA was denatured with 2 M HCl for 30 min after permeabilization. Images were acquired using a Leica TCS SP8 confocal microscope.

### PLA

Cells grown on the cover slides were treated with or without UVC irradiation 1.5 h before fixation and permeabilization each for 10 min at room temperature. The slides were blocked with 5% BSA for 30 min and incubated with primary antibodies for 2 h at room temperature. Then cells were subjected to proximity ligation assays using Duolink In Situ Red Starter kit (Sigma-Aldrich) according to the manufacturer’s protocol. Images were acquired using a Leica TCS SP8 confocal microscope.

### Cellular fractionation

The cytoplasmic and nuclear fractions were obtained as described^54^. Briefly, cells were lysed in 8 volume of hypotonic buffer (20 mM HEPES, pH 7.9, 10 mM KCl, 2 mM MgCl2, 0.3% Nonidet P-40, 1 mM DTT, 1 mM EDTA) supplemented with protease inhibitor cocktail (Sigma) on ice for 20 min. The lysate was centrifuged at 15,000× *g*, 4 °C for 20 min and the supernatant was retained as cytoplasmic proteins. The pellet was washed once with hypotonic buffer and resuspended in extraction buffer (20 mM HEPES, pH 7.9, 420 mM NaCl, 1 mM EDTA, 2 mM MgCl_2_, 10% glycerol, 1 mM DTT) containing protease inhibitor cocktail on ice for 20 min. After centrifugation at 15,000× *g*, 4 °C for 20 min, the supernatant was collected as nuclear proteins.

The chromatin fraction was obtained as described^49^ with modifications. Cells were lysed in buffer B (20 mM HEPES, pH 7.9, 150 mM NaCl, 0.5 mM MgCl_2_, 1 mM DTT, 0.5% Triton X-100, and 10% glycerol) supplemented with protease inhibitor cocktail on ice for 20 min. The lysate was centrifuged at 15,000 rpm, 4 °C for 20 min and the supernatant was removed. The pellet was washed once with buffer B and resuspended in 0.2 M HCl and extracted on ice for 20 min to solubilize the chromatin-bound proteins.

### Co-IP and western blotting assay

Cells were lysed in lysis buffer (50 mM Tris-HCl pH 7.4, 150 mM NaCl, 1% (v/v) NP-40, 1 mM EDTA, 1% sodium deoxycholate) containing protease inhibitor cocktail on ice for 30–40 min. The lysate was then centrifuged at 14,000 rpm, 4 °C for 15 min. A few aliquots of the supernatant were saved as Input and the remaining supernatant was incubated with a primary antibody overnight, followed by incubation with Pierce™ Protein G Agarose beads (Thermo Scientific™) for 1–2 h at 4 °C. The beads were washed with lysis buffer containing 150 mM, 300 mM and 450 mM NaCl, sequentially. The beads were boiled in loading buffer and subjected to SDS-PAGE and Western blotting analysis. For whole-cell protein extract, cells were incubated with lysis buffer supplemented with 0.1% SDS on ice for 30 min.

Chromatin co-IP was performed as described^55^ with some minor modifications. Cells were lysed in EBC-150 buffer (50 mM Tris-HCl, pH 7.5, 150 mM NaCl, 0.5% NP-40, 2 mM MgCl_2_) supplemented with protease inhibitor cocktails for 20 min at 4 °C, followed by centrifugation at 14,000 rpm (4 °C) for 15 min. The pellet containing chromatin fraction was solubilized in EBC-150 buffer with 500 U/mL Universal nuclease (Thermo Scientific) for 1 h at 4 °C under rotation. Then the NaCl concentration of the lysis buffer was adjusted to 300 mM, and lysates were incubated for another 30 min at 4 °C, followed by centrifugation (14,000 rpm, 4 °C, 15 min) to collect the soluble chromatin in the supernatant. The chromatin fraction was incubated with a primary antibody overnight, followed by incubation with Pierce™ Protein G Agarose beads (Thermo Scientific™) for 1–2 h at 4 °C. The beads were then washed 5 times with EBC-300 buffer (50 mM Tris-HCl, pH 7.5, 300 mM NaCl, 0.5% NP-40, 1 mM EDTA) and boiled in loading buffer, and the co-IP-precipitated proteins were subjected to SDS-PAGE and western blotting analysis.

### Statistical analysis

Statistical analyses were performed in GraphPad Prism 9.0 using unpaired Student’s *t*-test. Data in Fig. 5i, k were shown as means ± SD, and all the other data were shown as means ± SEM. A value of *P* < 0.05 is considered statistically significant (ns no significance, *P < 0.05, **P < 0.01, ***P < 0.001, ****P < 0.0001).

### Supplementary information

Supplementary Information

## References

[1] Marteijn JA, Lans H, Vermeulen W, Hoeijmakers JH (2014). Understanding nucleotide excision repair and its roles in cancer and ageing. Nat. Rev. Mol. Cell Biol..

[2] Shuck SC, Short EA, Turchi JJ (2008). Eukaryotic nucleotide excision repair: from understanding mechanisms to influencing biology. Cell Res..

[3] Reardon JT, Sancar A (2005). Nucleotide excision repair. Prog. Nucleic Acid Res. Mol. Biol..

[4] Sancar A (1996). DNA excision repair. Annu. Rev. Biochem..

[5] Cleaver JE (1968). Defective repair replication of DNA in xeroderma pigmentosum. Nature.

[6] Aboussekhra A (1995). Mammalian DNA nucleotide excision repair reconstituted with purified protein components. Cell.

[7] Mu D, Hsu DS, Sancar A (1996). Reaction mechanism of human DNA repair excision nuclease. J. Biol. Chem..

[8] Hanawalt P (1994). Transcrition-coupled repair and human disease. Science.

[9] Fousteri M, Mullenders LH (2008). Transcription-coupled nucleotide excision repair in mammalian cells: molecular mechanisms and biological effects. Cell Res..

[10] Lans H, Hoeijmakers JHJ, Vermeulen W, Marteijn JA (2019). The DNA damage response to transcription stress. Nat. Rev. Mol. Cell Biol..

[11] Hanawalt PC, Spivak G (2008). Transcription-coupled DNA repair: two decades of progress and surprises. Nat. Rev. Mol. Cell Biol..

[12] Hu J, Adar S, Selby CP, Lieb JD, Sancar A (2015). Genome-wide analysis of human global and transcription-coupled excision repair of UV damage at single-nucleotide resolution. Genes Dev..

[13] Liu J, Wu Z, He J, Wang Y (2022). Cellular fractionation reveals transcriptome responses of human fibroblasts to UV-C irradiation. Cell Death Dis..

[14] Djebali S (2012). Landscape of transcription in human cells. Nature.

[15] Geijer ME, Marteijn JA (2018). What happens at the lesion does not stay at the lesion: transcription-coupled nucleotide excision repair and the effects of DNA damage on transcription in cis and trans. DNA Repair.

[16] Boetefuer EL, Lake RJ, Fan HY (2018). Mechanistic insights into the regulation of transcription and transcription-coupled DNA repair by Cockayne syndrome protein B. Nucleic Acids Res..

[17] Wang W, Xu J, Chong J, Wang D (2018). Structural basis of DNA lesion recognition for eukaryotic transcription-coupled nucleotide excision repair. DNA Repair.

[18] Xu J (2017). Structural basis for the initiation of eukaryotic transcription-coupled DNA repair. Nature.

[19] Chiou YY, Hu J, Sancar A, Selby CP (2018). RNA polymerase II is released from the DNA template during transcription-coupled repair in mammalian cells. J. Biol. Chem..

[20] Tufegdžić Vidaković A (2020). Regulation of the RNAPII pool is integral to the DNA damage response. Cell.

[21] Nakazawa Y (2020). Ubiquitination of DNA damage-stalled RNAPII promotes transcription-coupled repair. Cell.

[22] Kokic G (2019). Structural basis of TFIIH activation for nucleotide excision repair. Nat. Commun..

[23] Mullenders L (2015). DNA damage mediated transcription arrest: step back to go forward. DNA Repair.

[24] Sarker AH (2005). Recognition of RNA polymerase II and transcription bubbles by XPG, CSB, and TFIIH: insights for transcription-coupled repair and Cockayne Syndrome. Mol. Cell.

[25] Groisman R (2006). CSA-dependent degradation of CSB by the ubiquitin-proteasome pathway establishes a link between complementation factors of the Cockayne syndrome. Genes Dev..

[26] Wei L (2011). BRCA1 contributes to transcription-coupled repair of DNA damage through polyubiquitination and degradation of Cockayne syndrome B protein. Cancer Sci..

[27] He J, Zhu Q, Wani G, Sharma N, Wani AA (2016). Valosin-containing protein (VCP)/p97 segregase mediates proteolytic processing of Cockayne Syndrome group B (CSB) in damaged chromatin. J. Biol. Chem..

[28] Schwertman P (2012). UV-sensitive syndrome protein UVSSA recruits USP7 to regulate transcription-coupled repair. Nat. Genet..

[29] Zhang X (2012). Mutations in UVSSA cause UV-sensitive syndrome and destabilize ERCC6 in transcription-coupled DNA repair. Nat. Genet..

[30] Liebelt F (2020). Transcription-coupled nucleotide excision repair is coordinated by ubiquitin and SUMO in response to ultraviolet irradiation. Nucleic Acids Res..

[31] Rosenzweig R, Nillegoda NB, Mayer MP, Bukau B (2019). The Hsp70 chaperone network. Nat. Rev. Mol. Cell Biol..

[32] Zou Y, Crowley DJ, Van Houten B (1998). Involvement of molecular chaperonins in nucleotide excision repair. Dnak leads to increased thermal stability of UvrA, catalytic UvrB loading, enhanced repair, and increased UV resistance. J. Biol. Chem..

[33] Kwon SB (2002). Impaired repair ability of hsp70.1 KO mouse after UVB irradiation. J. Dermatol. Sci..

[34] Roh BH, Kim DH, Cho MK, Park YL, Whang KU (2008). Expression of heat shock protein 70 in human skin cells as a photoprotective function after UV exposure. Ann. Dermatol..

[35] Moriel-Carretero M, Tous C, Aguilera A (2011). Control of the function of the transcription and repair factor TFIIH by the action of the cochaperone Ydj1. Proc. Natl. Acad. Sci. USA.

[36] Huang Y (2023). DNAJA2 deficiency activates cGAS-STING pathway via the induction of aberrant mitosis and chromosome instability. Nat. Commun..

[37] Prosser SL (2022). Aggresome assembly at the centrosome is driven by CP110-CEP97-CEP290 and centriolar satellites. Nat. Cell Biol.

[38] An J (2011). Strand-specific PCR of UV radiation-damaged genomic DNA revealed an essential role of DNA-PKcs in the transcription-coupled repair. BMC Biochem..

[39] Mellon I, Rajpal DK, Koi M, Boland CR, Champe GN (1996). Transcription-coupled repair deficiency and mutations in human mismatch repair genes. Science.

[40] Mellon I, Bohr VA, Smith CA, Hanawalt PC (1986). Preferential DNA repair of an active gene in human cells. Proc. Natl Acad. Sci. USA.

[41] Simon TJ, Smith CA, Friedberg EC (1975). Action of bacteriophage T4 ultraviolet endonuclease on duplex DNA containing one ultraviolet-irradiated strand. J. Biol. Chem..

[42] Kaushik S, Cuervo AM (2018). The coming of age of chaperone-mediated autophagy. Nat. Rev. Mol. Cell Biol..

[43] Wu Y, Zhang J, Fang L, Lee HC, Zhao YJ (2019). A cytosolic chaperone complex controls folding and degradation of type III CD38. J. Biol. Chem..

[44] Walker VE (2010). Hsp40 chaperones promote degradation of the HERG potassium channel. J. Biol. Chem..

[45] Baaklini I, Gonçalves CC, Lukacs GL, Young JC (2020). Selective binding of HSC70 and its co-chaperones to structural hotspots on CFTR. Sci. Rep..

[46] Nakazawa Y (2012). Mutations in UVSSA cause UV-sensitive syndrome and impair RNA polymerase IIo processing in transcription-coupled nucleotide excision repair. Nat. Genet..

[47] Sin Y, Tanaka K, Saijo M (2016). The C-terminal region and SUMOylation of Cockayne Syndrome group B protein play critical roles in transcription-coupled nucleotide excision repair. J. Biol. Chem..

[48] Pines A (2018). TRiC controls transcription resumption after UV damage by regulating Cockayne syndrome protein A. Nat. Commun..

[49] Lake RJ, Geyko A, Hemashettar G, Zhao Y, Fan HY (2010). UV-induced association of the CSB remodeling protein with chromatin requires ATP-dependent relief of N-terminal autorepression. Mol. Cell.

[50] Lake RJ, Fan HY (2013). Structure, function and regulation of CSB: a multi-talented gymnast. Mech. Ageing Dev..

[51] Park C, Suh Y, Cuervo AM (2015). Regulated degradation of Chk1 by chaperone-mediated autophagy in response to DNA damage. Nat. Commun..

[52] Shalem O (2014). Genome-scale CRISPR-Cas9 knockout screening in human cells. Science.

[53] Holcomb N (2017). Inorganic arsenic inhibits the nucleotide excision repair pathway and reduces the expression of XPC. DNA Repair.

[54] Enokido Y (2010). Mutant huntingtin impairs Ku70-mediated DNA repair. J. Cell Biol..

[55] van den Heuvel D (2021). A CSB-PAF1C axis restores processive transcription elongation after DNA damage repair. Nat. Commun..

