# Supplementary material for: Heat shock protein DNAJA2 regulates transcription-coupled repair by triggering CSB degradation via chaperone-mediated autophagy
Source: Cell Discov. 2023 Oct 31;9:107. doi: 10.1038/s41421-023-00601-8 (PMC10618452; doi:10.1038/s41421-023-00601-8)
Supplement: Supplementary file 1 — Supplementary Information [file 41421_2023_601_MOESM1_ESM.pdf]

Supplementary Information

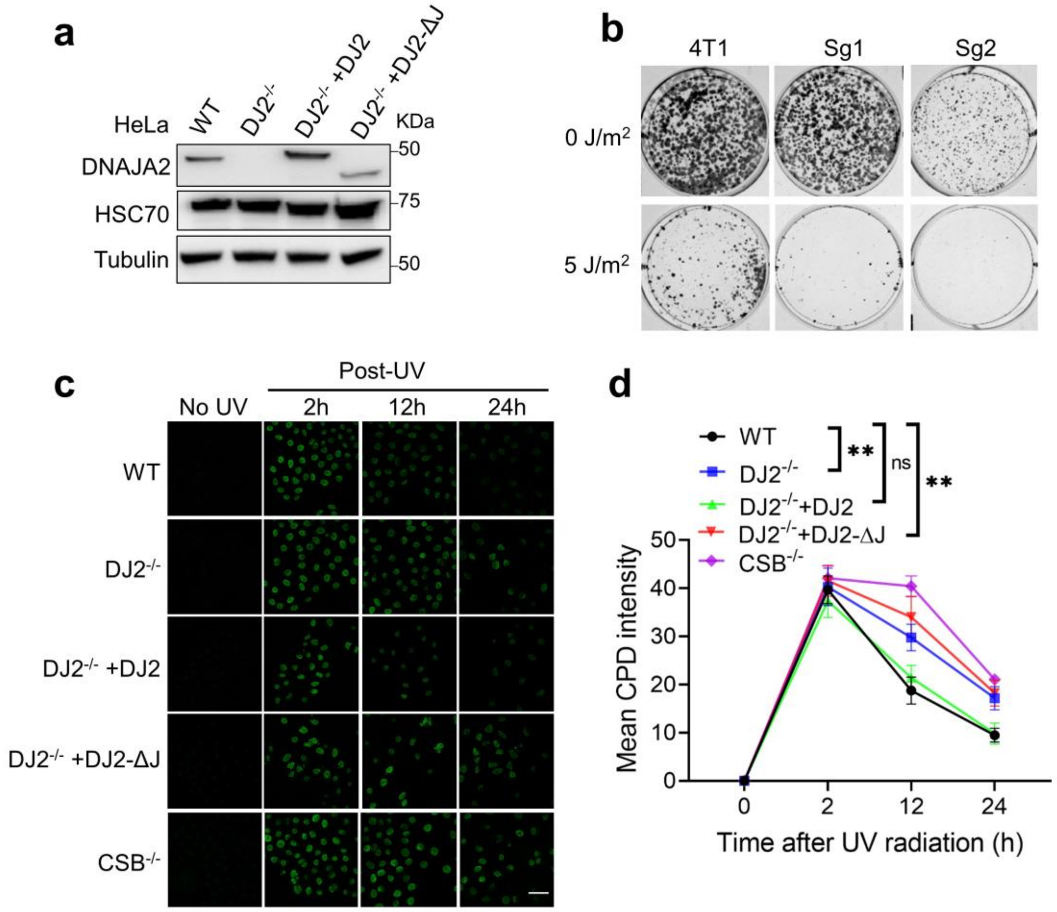

Supplementary Fig. S1: DNAJA2 is required for UV damage repair, related to Fig. 1. a Western blotting analysis showing the expected expression of various forms of DNAJA2 in HeLa WT, *DNAJA2* knockout ($DJ2^{-/-}$) and knockouts rescued with WT *DNAJA2* ($DJ2^{-/-}$+DJ2) or J-domain truncated *DNAJA2* ($DJ2^{-/-}$+DJ2-ΔJ). **b** Colony formation assay showing UV hypersensitivity of two 4T1-*DNAJA2*-knockout clones (Sg1 and Sg2). **c** Immunofluorescence analysis showing CPD levels in WT, $DJ2^{-/-}$, $DJ2^{-/-}$+DJ2 and $DJ2^{-/-}$+DJ2-ΔJ HeLa cells at different time points after UV treatment (5 J/m^2) . $CSB^{-/-}$ cells were used as a control. **d** Quantifications of mean CPD intensity, as shown in (**c**). Data of three independent experiments were used for the quantification and statistical analysis. Scale bar, 50 μm. *P* values were determined by two-tailed unpaired t test. ns, $P > 0.05$; $^{**}P < 0.01$.

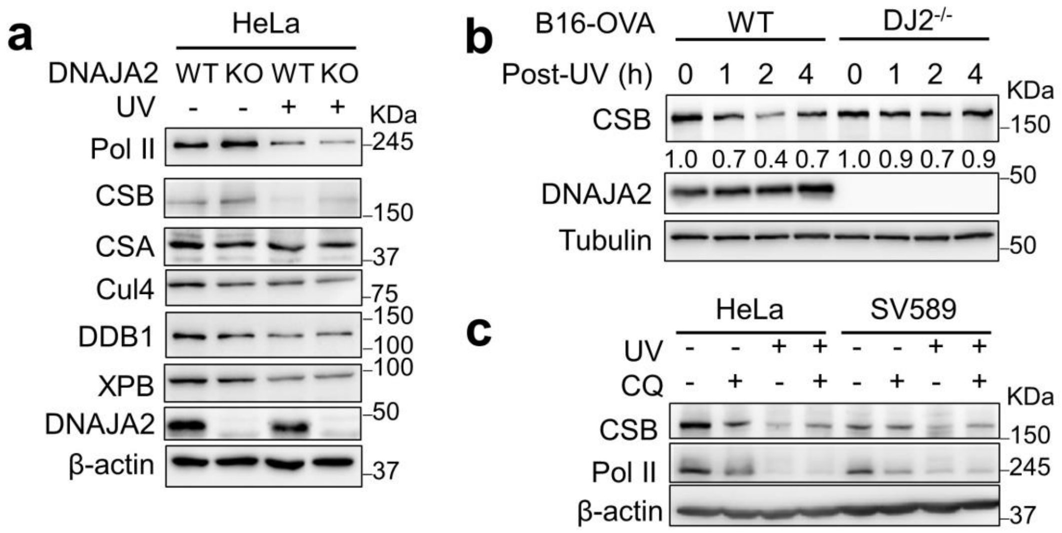

Supplementary Fig. S2: DNAJA2 is required for lysosome-conducted CSB degradation upon UV irradiation, related to Fig. 2. a Western blots showing the expression of several key TC-NER factors in WT and DJ2$^{-/-}$ HeLa cells without UV treatment or 2h post-UV treatment. **b** Western blots showing the protein stability of CSB after UV treatment in WT and DJ2$^{-/-}$ B16-OVA cells. **c** Western blots showing the protein stability of CSB and Pol II upon UV treatment in the presence of a lysosomal inhibitor CQ in HeLa and SV589 cells.

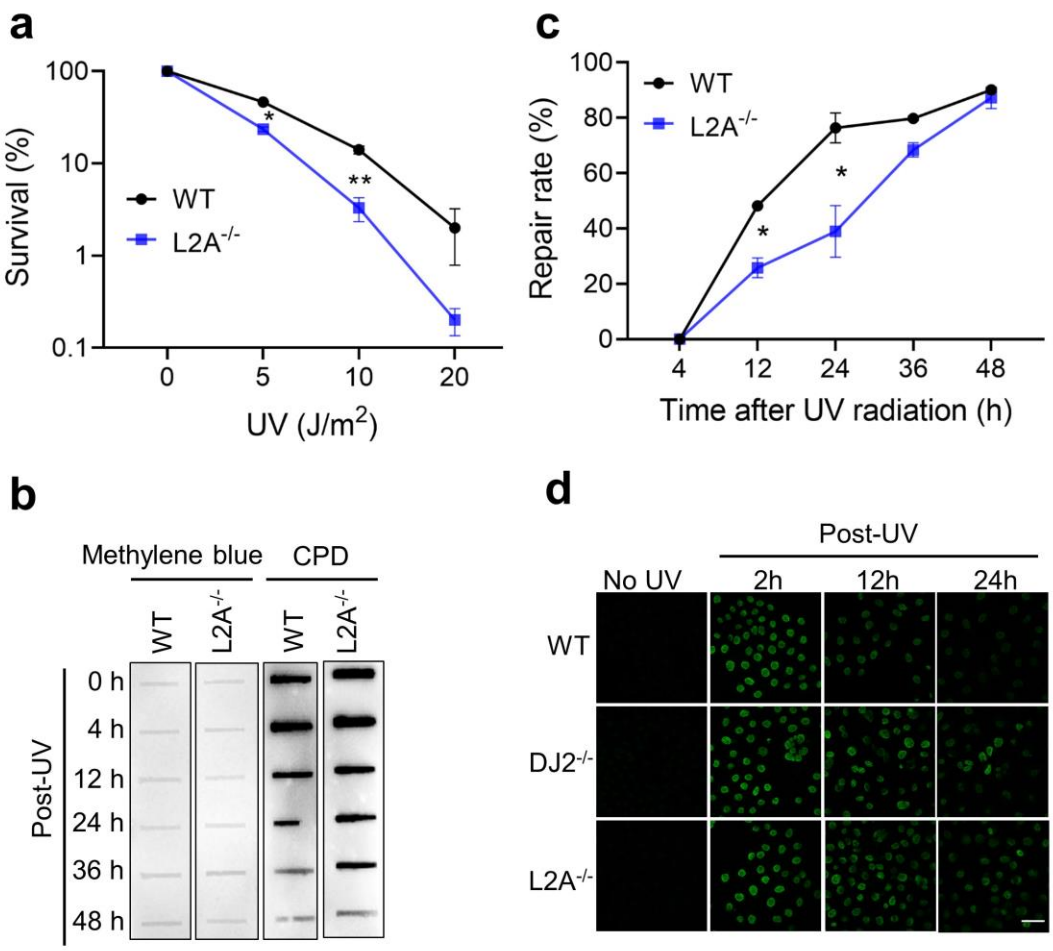

Supplementary Fig. S3: CMA-deficient cells are defective in UV damage response and repair, related to Figure 3. a Colony formation assay showing surviving fractions of WT and L2A$^{-/-}$ HeLa cells treated with different doses of UV, as indicated. **b** Slot blots showing CPD levels using an anti-CPD antibody in WT and L2A$^{-/-}$ HeLa cells at different time points post-UV irradiation (5 J/m^2). Methylene blue staining shows the total DNA loaded in the assay. **c** Quantification of the UV lesion repair efficiency (%) in WT and L2A$^{-/-}$ HeLa cells at different time points after UV irradiation. CPD repair was calculated as: Repair (%) = (1 - CPD level of a given hour post UV/CPD level of 0 hour post UV) 100%. Data from three independent experiments were used for the quantification and statistical test. **d** Immunofluorescence analysis showing CPD intensity before or after UV irradiation (5 J/m^2) in WT, DJ2$^{-/-}$ and L2A$^{-/-}$ HeLa cells as indicated. Scale bar, 50 µm. *P* values were determined by two-tailed unpaired t test. **P* < 0.05; ***P* < 0.01.

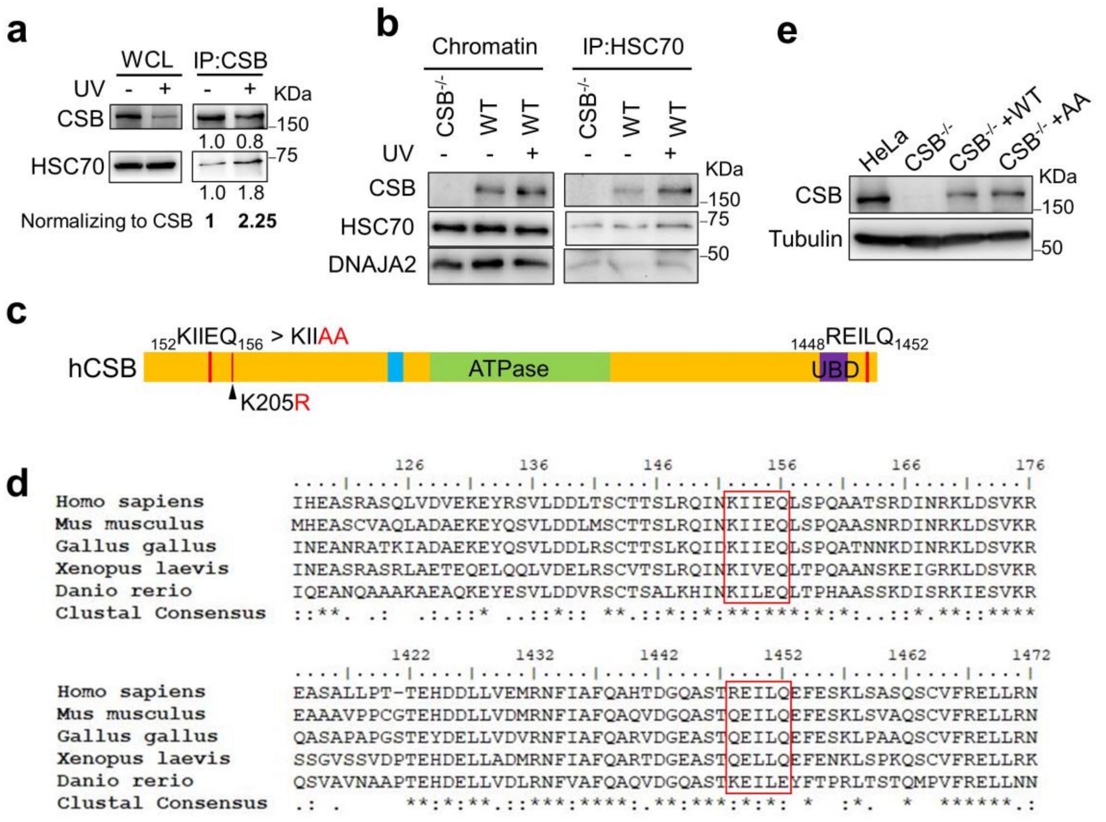

Supplementary Fig. S4: UV treatment induces CSB degradation via CMA pathway, related to Fig. 3, 4. a, b Co-IP Western analyses showing that UV irradiation promotes the interaction between CSB and HSC70 in both whole-cell lysates (**a**) and chromatin fractions (**b**). **c** Schematic diagram showing locations of two canonical KFERQ-like motifs (thick red bars) and K205 (thin red bar) residue in human CSB protein. The KFERQ-mutated CSB used in this study contains EQ>AA substitutions at the N-terminus. **d** Amino acid sequence alignment of CSB proteins from different species. The two KFERQ-like motifs are highlighted by red rectangles. **e** Western blotting analysis showing the expected expression of various forms of CSB in HeLa WT, *CSB* knockout (CSB$^{-/-}$) and knockouts rescued with WT CSB (CSB$^{-/-}$+WT) or AA-CSB (CSB$^{-/-}$+AA).

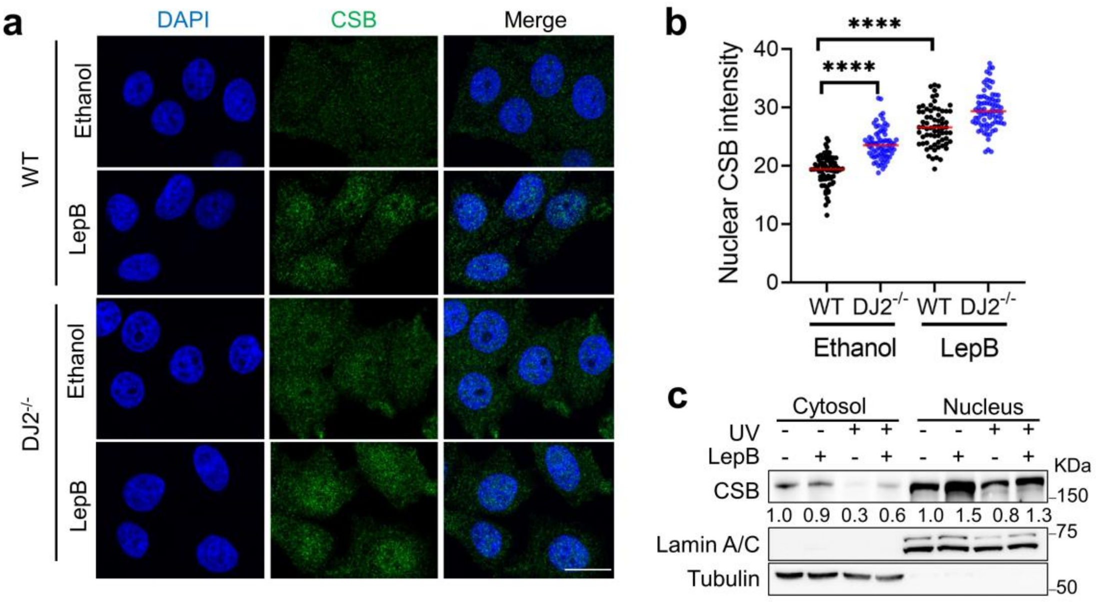

Supplementary Fig. S5: Nuclear export inhibition blocks UV-induced CSB degradation, related to Fig. 5. a Immunofluorescence analysis showing the CSB intensity 2h post 20 J/m^2 UV irradiation in WT and DJ2$^{-/-}$ HeLa cells treated with or without LepB. Cells were pre-treated with 20 nM LepB for 6h before UV treatment. **b** Quantification of the CSB intensity (n = 69-77), as shown in (**a**). **c** Western blots showing CSB degradation upon UV irradiation in cytosolic and nuclear fractions in the presence or absence of LepB treatment. Cells were pre-treated with or without 20 nM LepB for 6h before UV treatment (20 J/m^2) and continuously cultured for 2h in the presence or absence of LepB. Scale bar, 20 μm. *P* values were determined by two-tailed unpaired t test. *****P* < 0.0001.
